# Curriculum management/monitoring in undergraduate medical education: a systematized review

**DOI:** 10.1186/s12909-019-1495-0

**Published:** 2019-02-19

**Authors:** Tahereh Changiz, Nikoo Yamani, Shahram Tofighi, Fatemeh Zoubin, Batool Eghbali

**Affiliations:** 1 Medical Education Research Center, Isfahan University of Medical Sciences, Isfahan, Iran; 2 Health Management Research Center, Baqiyatallah University of Medical Sciences, Tehran, Iran

**Keywords:** Undergraduate Medical Education (UME), Curriculum management, Curriculum monitoring, Systematized review

## Abstract

**Background:**

Monitoring and management of undergraduate medical education (UME) curricula are crucial contributors to successful medical education. This systematized review explores the different approaches that medical schools have to UME curriculum management or monitoring in order to provide a basis for curriculum managers.

**Methods:**

PubMed, Science Direct, Scopus, and ERIC were searched with no time limitation using the keywords *curriculum*, *medicine, management, monitoring*, and *alignment*. Advanced search options and Boolean operators ‘AND’ and ‘OR’ were also used to find more relevant records.

**Results:**

From a total of 673 records, 14 articles along with 7 papers from hand searching and snowballing were included in the review. Documents were categorized into 3 groups of UME curriculum management: developing computerized tools, surveying curriculum stakeholders and reviewing curriculum documents, and introducing managerial structures.

**Conclusions:**

Different approaches are reported for UME curriculum management/monitoring at different levels. Managerial structures and computerized tools are most frequently used at the college level because of the large number of faculty members who are responsible for the UME curriculum delivery and the large amount of complex curriculum information. Surveys and reviews of curriculum documents are used mostly to manage a part of a UME curriculum or to monitor teaching of a certain subject during all or some of the educational years.

## Background

A curriculum is the foundation of any educational institution [1], and the quality of its implementation can be a good indicator of the institution’s educational efficacy. To ensure proper curriculum implementation, the curriculum should be managed carefully. There are different definitions for *curriculum management*; it is sometimes used as a synonym for ‘teaching and learning management’ or as equal to ‘managing the whole institution’ [2]. According to Stansbury and Huenecke [3], it is a process with four main phases: identifying and establishing goals; determining a process for guiding educational specialists to attain these goals; establishing managerial techniques for implementation of the identified process; and constant evaluating and reevaluating the identified goals, processes, and managerial techniques. Another relevant term in this field is *curriculum monitoring*. Monitoring is a process based on clear aims and is performed to assess the quality of work, to show which targets and standards are achieved and which are not, and to clarify where improvement is needed [4]. Furthermore, the term *curriculum evaluation* refers to the description and judgment about the value or worth of the curriculum plans, processes, and outcomes to provide evidence to inform decision-makers [5]. It answers if the planned and implemented objectives have been met [6]. Although monitoring and evaluation are sometimes used as synonyms, there are some differences. *Monitoring* both looks forward and backward and tries to answer the question ‘Are we reaching there?’. It is a regular continuous and systematic process that collects data routinely to correct and improve the program through feedback, while evaluation is often periodic and has a backward view to answer the question ‘Have we reached there?’. The data collected on curriculum monitoring can be used as part of the evidence for curriculum evaluation. Both monitoring and evaluation can fulfill the informational requirements for curriculum management [4, 5]. Because of their dynamic characteristics, the focus of this paper is on curriculum management and monitoring.

Managing and monitoring the curriculum for undergraduate medical education (UME), due to its complexity and importance in the healthcare system, have been considered in curriculum planning and development. Kern [7] introduced a six-step approach to medical curriculum development where the sixth step is ‘evaluation and feedback’; this step helps curriculum improvement and prepares evidence of curriculum efficacy. In another approach, Harden [8] considered 10 steps for curriculum planning and evaluation. Attention to the last step, i.e., managing the curriculum, has gained more importance because of reasons such as increasing complexity of the curriculum and changes in the healthcare system and medical practice [8]. On the other hand, accrediting bodies including the Liaison Committee on Medical Education (LCME) [9] and the World Federation for Medical Education (WFME) [10] have established standards for UME curriculum management and monitoring. In this regard, several medical schools have made efforts in documenting, monitoring, and managing their educational performance with regard to their respective UME curriculum to meet standards [11].

However, there are differences between these reports, most significantly, as concerns with their focus of inquiry. For example, in O’Brain’s study [12], deans of medical schools and individuals interested in healthcare-associated infections (HCAI) in medical schools participated in a survey about the content and the teaching and assessment methods related to the HCAI subject. Elsewhere, computerized tools such as CurrMIT (Curriculum Management & Information Tool) [13] and eMed [14] were designed and implemented to manage UME curricula. Documenting these diversities and their different aspects can be useful for UME curriculum managers in planning more suitable approaches to UME curriculum management or monitoring.

In this paper, curriculum management or monitoring denotes every activity to address concerns about the whole or part of a UME curriculum delivery (how, when, and what is taught and assessed). To find these activities, we searched online data sources to describe the different aspects of UME curriculum management in medical schools. This review was designed to address the following questions:

- What experiences have been reported in the field of UME curriculum management or monitoring?
- What is the focus of these experiences?

## Methods

This is a systematized review, which is similar to a systematic review except for the fact that it fails to contain some of the systematic review components [15]. PubMed, Science Direct, Scopus, and ERIC were searched as electronic data sources to find the reported studies on UME curriculum management or monitoring.

With regard to the research questions, the following keywords were used to search through these online data sources with no time limitation: curriculum, medicine, management, monitoring, and alignment. Advanced search options and Boolean operators ‘AND’ and ‘OR’ were also used to find out more relevant records. Table 1 shows the search strategies in more detail.

**Table 1:** Search strategy details

Search Engine/ Database	Search strategy	Date of search
PubMed	((curricul* [Title]) AND (manage* OR monitor* OR alignment [Title]) AND (medic* [Title]))	24.7.2017
Science Direct	TITLE-ABSTR-KEY((curricul*) AND (manage* OR monitor* OR align*) AND (medic*)) AND LIMIT-TO(topics, “medical, medical education, medical student, medicine”).	1.8.2017
Scopus	TITLE ((curricul*) AND (manage* OR monitor* OR align*) AND (medic*))	31.7.2017
ERIC	(curriculum or curricula) AND (manage* OR monitor* OR control* OR alignment) AND (medicine OR medical) AND (Descriptor: Medical Education)	29.7.2017

To select appropriate papers for data collection, all the records were saved and checked for duplication. After removing duplicate records, the titles were screened in terms of eligibility criteria. Records with definitely irrelevant titles were excluded and those with (potentially) relevant titles were saved for abstract screening. Uncertain and relevant abstracts were selected for full-text assessment. Full-texts were assessed carefully to find eligible articles. In addition, appropriate papers from hand searching and snowballing were included in the review. We used Mendeley Desktop version 1.17.6 as a reference manager tool.

The following items were set as the eligibility criteria for inclusion of papers in this review:

- Paper format: Journal article
- Paper language: English
- Paper subject: Curriculum management or monitoring in the field of UME (curriculum as a whole or a smaller part of it)

To exclude non-relevant papers, these items were considered:

- Paper format: Anything other than a journal article (book or book section, conference abstract, etc.)
- Paper subject:
  - Curriculum management or monitoring in fields other than UME
  - Pure reports of curriculum evaluation, change, or development
  - Papers with a curriculum management claim but not truly reporting curriculum management/monitoring

There are no ethical issues applicable in this study.

## Results

**Fig. 1:**
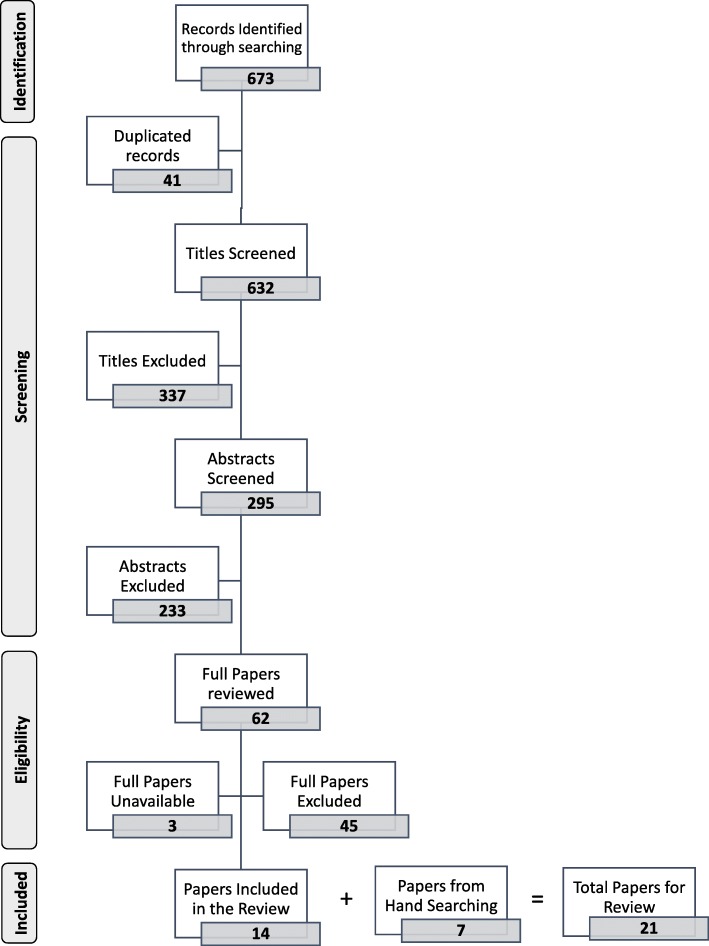
PRISMA flowchart for including papers in the review

In total, 673 records were identified by searching 4 online data sources. After removing duplicate records and records with irrelevant titles, abstracts, or full papers, 14 articles were selected for data extraction. In addition, 7 articles retrieved from hand searching and snowballing were added to the review. The selection process to include papers in the review is displayed in Fig. 1.

The selected papers had different foci, which are categorized into three groups of UME curriculum management or monitoring, as summarized in Table 2.

**Table 2:** Classification of articles

Classification	Articles
Developing Computerized Tools	Rosinski E. 1962 [16], Gotlib D. 1984 [17], Curry L. 1984 [18], Buckenham S. 1986 [19], Mattern W. 1992 [20], Salas A. 2003 [13], Watson E. 2007 [14], Nifakas S. 2015 [21], Balzer F. 2015 [22], Stekette C. 2015 [23], Shroyer A. 2016 [11], Al-Eyd 2018 [24]
Surveying Curriculum Stakeholders and Reviewing Curriculum Documents	Maccromick R. 1992 [25], Atienza M. 2007 [26], Van Aalst-Cohen E. 2008 [27], O’Brien D. 2009 [12]
Introducing Managerial Structure	Silber D. 1978 [28], Davis W.K. 1993 [29], Harden R.M. 1997 [30], Fong S. F. 2015 [31], Klement B. 2017 [32]

*Developing computerized tools* was reported in 12 papers; these tools were used to facilitate the UME curriculum management or monitoring process at different levels (Table 3)

**Table 3:** Articles classified under ‘Developing Computerized Tools’ category

First Author. Year	Title	Site of Study	Summary
Rosinski E. 1962 [16]	A System of Cataloguing the Subject Matter Content of a Medical School Curriculum	Medical College of Virginia	The subjects that were taught during 4 years of medical school were cataloged in a computer and could be searched by a unique code for each subject. This system was helpful for medical teachers to locate the searched subject throughout the four–year medical curriculum.
Gotlib D. 1984 [17]	A computerized database-management system for curriculum analysis	University of Ottawa Medical School	A computer-based information system was designed to help the curriculum committee have quick and simple access to the data needed for management decisions related to *pathophysiology and special pathology* course.
Curry L. 1984 [18]	Computerization of undergraduate medical curriculum content	Dalhousie university, Faculty of Medicine	Summaries of the first 3 years of undergraduate medical curriculum were stored in a computerized system. Each teaching group had a global access to these summaries; hence, unnecessary redundancies could be avoided.
Buckenham S. 1986 [19]	An Application of Computers to Curriculum Review and Planning	University of Toronto Faculty of Medicine	A computer-based curriculum database was developed for undergraduate medical curriculum (except the clerkship year) to provide accurate curriculum information for curriculum planners.
Mattern W. 1992 [20]	Computer databases of medical school curricula	–	Description of 3 medical curriculum database prototypes: University of North Carolina at Chapel Hill SoM: first 2 years of the curriculum University of Maryland: data on core elements of the first 3 years of the curriculum University of Miami SoM: preclinical curriculum
Salas A. 2003 [13]	CurrMIT: a tool for managing medical school curricula	North American Medical Schools	To document and manage the curriculum information of North American medical schools, CurrMIT was developed and implemented by the AAMC (Association of American Medical Colleges)
Watson E. 2007 [14]	Development of eMed: A Comprehensive, Modular Curriculum-Management System	University of New South Wales Faculty of Medicine	An electronic curriculum-management system (eMed) was developed to support the development and delivery of new undergraduate medical curriculum. It contains 6 main tools: curriculum map, timetable tool, course management tool, learning resource catalog, student portfolio, assessment tracking tool, and peer assessment tool.
Nifakas S. 2015 [21]	AUVA – Augmented Reality Empowers Visual Analytics to explore Medical Curriculum Data	Year 3 undergraduate medical program at Karolinska Institutet	Technical description of using Augmented Reality (AR) as a data management-presentation tool to visualize medical curriculum data.
Balzer F. 2015 [22]	Development and alignment of undergraduate medical curricula in a web-based, dynamic Learning Opportunities, Objectives and Outcome Platform (LOOOP)	Fourth-year students of an organ-based, interdisciplinary curriculum of human medicine, participated in the study	*Curriculum map* and *Learning Opportunities, Objectives and Outcome Platform* (LOOOP) were developed to ensure alignment of different parts of the medical curriculum (competencies, objectives, teaching and assessment methods, etc.).
Stekette C. 2015 [23]	Prudentia: A Medical School’s Solution to Curriculum Mapping and Curriculum Management	School of Medicine University of Notre Dame Australia	In response to the accreditation visits by the Australian Medical Council (AMC), a curriculum mapping system (Prudentia) was designed and developed to demonstrate alignment between different curriculum components such as course outcomes, AMC outcomes, etc.
Shroyer A. 2016 [11]	Drivers of dashboard development (3-D): A curricular continuous quality improvement approach	Stony Brook University School of Medicine	To meet LCME accreditation standards, Drivers of Dashboard Development (3-D) approach was developed and implemented to collect and monitor UME program data.
Al-Eyd G. 2018 [24]	Curriculum mapping as a tool to facilitate curriculum development: a new School of Medicine experience	California University of Science & Medicine- School of Medicine (CalMed-SOM)	Introduce the process of curriculum mapping using the standardized curriculum inventory vocabulary to align the medical school to the AAMC. This was done through a computerized data collection tool called Session Mapping Template (SMT).

Although there are minor differences between the tools, the common feature presented in these articles is providing relevant and accessible information for the various stakeholders involved in UME curricula (curriculum planners, administrators, medical teachers, and students), which can be used to monitor or manage the curricula. From a historical perspective, computerized tools were first designed to provide a searchable repository of the large UME curricula data, which was mainly used to locate specific coded subjects and to define gaps and redundancies across the stored data [16–20]. These tools were used to facilitate curriculum-related decision-making [16, 17, 19] and curriculum information management [18, 20] at the level of UME curricula as a whole [16], part of the UME curricula [18–20], or a specific course [17]. In a more expanded scope, CurrMIT was developed by the Association of American Medical Colleges (AAMC) to support the North American medical schools in UME curriculum monitoring and management. The proprietary feature of the CurrMIT for each medical school was the ability to view other schools’ programs and to learn from their experiences [13]. A comprehensive modular system, eMed, was designed and implemented at the University of New South Wales Faculty of Medicine to support the development and delivery of its new 6-year undergraduate medicine program. This system with its six main tools could support students and teachers in the learning and teaching process in addition to supporting curriculum managers in administrative processes [14]. Nifakas [21] reported on how to use Augmented Reality (AR) technology as a data management and presentation tool for visualizing UME curriculum data. Presenting a schematic view of the non-taught, non-examined, and examined but non-taught learning outcomes make it very easy for curriculum managers to have a rapid overview of what is happening in the curriculum. This could be used for curriculum monitoring. Recent articles have highlighted the role of accreditation in developing computerized tools for UME curriculum management or monitoring [11, 22–24]. These tools have been used to manage UME curricula through aligning different parts of the curriculum [22–24] and monitoring UME program data [11].

*Surveying curriculum stakeholders and reviewing curriculum documents* were used in 4 studies to collect the data needed for curriculum management-related activities. Table 4 displays summaries of these papers.

**Table 4:** Articles classified under ‘Surveying Curriculum Stakeholders and Reviewing Curriculum Documents’ category

First Author. Year	Title	Site of Study	Summary
Maccromick R. 1992 [25]	A review of the oncology curriculum at Dalhousie medical school	Dalhousie medical school Oncology Curriculum	To determine deficiencies in the oncology curriculum, a survey of 30 department heads (description of what oncology topics and how they were taught) and recent graduate interns (their opinions of various aspects of teaching oncology) was conducted.
Atienza M. 2007 [26]	Development of a core curriculum on tuberculosis control for Philippine medical schools	Philippine medical schools	Monitoring evaluation of the TB control-DOTS core curriculum was conducted 10 months after implementation through a survey of administrators, project implementers, and faculty members who taught TB. In addition, curriculum documents (records, course outlines, syllabi, teaching-learning resources and activities, and assessment tools) were reviewed, and key informants were interviewed.
Van Aalst-Cohen E. 2008 [27]	Palliative care in medical school curricula: a survey of United States medical schools	U.S. Medical Schools	To identify how palliative care is incorporated in U.S. medical schools curricula, a survey of deans or their designees of all 128 allopathic U.S. medical schools was conducted; in addition, corresponding information was gathered from CurrMIT.
O’Brien D. 2009 [12]	Survey of teaching/learning of healthcare-associated infections in UK and Irish medical schools	Medical schools in the UK and the Republic of Ireland	To determine how healthcare-associated infection (HCAI) is taught and assessed, the deans of all the medical schools and individuals in the medical schools who were known to have an interest in HCAI, were invited to participate in a survey.

In one of these studies, a survey of 30 department heads and recent graduates was conducted at Dalhousie Medical School to determine how the oncology curriculum was implemented compared to an ideal one and to make appropriate decisions to improve students’ expertise in dealing with cancer [25]. A number of other studies were performed on a national scale. Atienza [26] reported a monitoring evaluation of the TB (tuberculosis) control-DOTS (Directly Observed Treatment Short-course) core curriculum through a survey of curriculum stakeholders and a review of curriculum documents. Following LCME requirements for teaching palliative care in accredited U.S. medical schools, a survey of 128 allopathic U.S. medical schools was conducted and related information from CurrMIT was gathered to find how the palliative care was addressed in the UME curriculum [27]. Considering the important role of medical doctors in the diagnosis, management, and prevention of HCAI, a survey of all medical schools in the UK and the Republic of Ireland was conducted to determine how HCAI was taught and assessed for medical students. The results of this study were used to assess the status quo and make decisions to improve the current condition [12].

In 5 cases, the authors introduced *managerial structures* (for example, curriculum committee) that were responsible for UME curriculum monitoring or management (Table 5).

**Table 5:** Articles classified under ‘Introducing Managerial Structure’ category

First Author. Year	Title	Site of Study	Summary
Silber D. 1978 [28]	The SIU medical curriculum: systemwide objectives-based instruction	Southern Illinois University School of Medicine	This paper is a description of an objective-based instructional system, which contains some specific procedures to monitor, maintain and improve the program. For example, the Student Progress Committee is responsible to certify student achievement of the desired objectives. In addition, a curriculum evaluation system is developed to monitor program implementation (defining needed modifications, supervising the implementation of these modifications, and assessing effects of this implementation) based on data collected at all levels of the curriculum.
Davis W.K. 1993 [29]	Centralized decision making in management of the curriculum at the University of Michigan Medical School	University of Michigan Medical School	This article describes how the managerial structure for MD curriculum management at Michigan Medical School has changed from a decentralized to a centralized format and explains both of these structures.
Harden R.M. 1997 [30]	The new Dundee medical curriculum: a whole that is greater than the sum of the parts	University of Dundee	Authors introduce the new Dundee medical curriculum, its philosophy and implementation. They emphasize the importance of curriculum committees and working groups in curriculum implementation. The structure of the Undergraduate Medical Education Committee and its responsibility to the Board of the Faculty of Medicine and Dentistry are described in this article.
Fong S. F. 2015 [31]	Liaison Committee on Medical Education Accreditation, Part III: Educational Program Content, Curriculum Management, and Student Assessment	John A. Burns School of Medicine at the University of Hawai‘i at Manoa (JABSOM)	This paper is a report of how LCME accreditation standards related to educational program content, curriculum management, and student assessment are addressed at JABSOM. The JABSOM Curriculum Committee programs and some institutional practices and procedures are introduced as the designed strategies to meet the elements of LCME Standard 8: “Curriculum Management, Evaluation and Enhancement”.
Klement B. 2017 [32]	Implementation and Modification of an Anatomy-Based Integrated Curriculum	Morehouse School of Medicine First-year curriculum	This article is a report of restructuring first-year medical curriculum from a discipline-based to an integrated program. In this regard, a curriculum management team was organized to deliver and manage the new curriculum efficiently. The role of each team member in addition to team performance is described in detail.

One of these studies described the instructional system in Southern Illinois University School of Medicine for a 3-year medical curriculum and the procedures to monitor, maintain, and improve the curriculum. In this regard, a curriculum evaluation system was developed to monitor program implementation through collecting data at all levels of the curriculum; the collected data were used for curriculum-related decision-making [28]. Managerial structures introduced in the other 4 papers of this category were established to manage the UME curriculum as a whole [29–31] or a specific course within the curriculum, e.g., anatomy [32]. In some cases, these structures were defined in response to accreditation requirements [31].

## Discussion

This systematized review was conducted to identify the reported experiences of managing or monitoring UME curricula. The selected articles addressed concerns with UME curriculum delivery (how, when, and what is taught and assessed) for different parts of the curriculum (course [12, 17, 26], one part of the UME curriculum [20, 21, 32], or the UME curriculum as a whole [16, 28, 31]) at various managerial levels of the medical school [16, 18, 19, 22, 28] or on national scales [12, 13]. Some of these concerns related to the large amount of curricular data in UME (diversity and multiplicity of learning objectives) [21], gaps and overlaps of the topics taught in different courses [16], information needed about subjects that were taught to medical students and their location through the curriculum [27], and addressing accreditation standards for medical schools [11, 23, 31]. These papers were classified based on their foci related to UME curriculum management or monitoring.

The 1st category consisted of papers that report designing and implementing computerized tools to facilitate managing or monitoring the UME curricula. The first computerized system for UME curriculum management was developed in the early 1960s [33]. It was designed at the Medical College of the Virginia School of Medicine with a simple feature of searching cataloged subjects throughout a 4–year medical curriculum [16]. With the advent of computer-based technology and worldwide application of computers, electronic contents, and digital libraries, more comprehensive computer tools were employed to improve informational provision for managerial decision-making (13,14). At a national level, CurrMIT was designed and implemented by AAMC as a centralized system to manage all North American medical schools’ curricula [13]. However, it failed to meet the informational needs of curriculum managers in nearly 200 medical schools, whereby many schools designed their own systems to address their local needs. In this regard, AAMC decided to develop the Curriculum Inventory Standard [13, 34] in cooperation with MedBiquitous to facilitate the representation of diverse curricula using a shared data model [34].

There are some other systems termed Learning Management System (LMS) and Learning Content/Curriculum Management System (LCMS). LMS focuses on students’ learning process and progression and LCMS has some additional functions related to content and course management [35]. Although Curriculum Management System (CMS) has some similarities to LMS or LCMS, it is generally applied in a more extended domain and focuses on the curriculum information required for managerial decision-making. Computers in any CMS are usually used as a repository for curriculum data, which can be searched and retrieved by users (administrators, faculty members, students, etc.) [13, 23]. The demand seems to be growing for more dynamic systems to deliver timely and automated feedback that can facilitate more efficient curriculum management at different levels.

The 2nd category comprised of research projects in which curriculum stakeholders were surveyed and curriculum documents were reviewed to gather data needed for UME curriculum management or monitoring. Hendricson [36] conducted a survey of 144 North American Medical Schools to identify different aspects of UME curriculum committees. In this study, medical schools with a systematic procedure for course review reported that they had used different processes, including interviews with faculty members and students and peer review of documents (i.e., syllabi, tests, and lectures) [36]. These methods are similar to some data collection methods used in program evaluation [37]; the collected data was used for managerial decision-making. Therefore, curriculum monitoring can be considered as a type of formative evaluation of the curriculum (as a program) in the sense of providing appropriate information for managerial decision-making regarding curriculum improvement.

The last category concerned with introducing the managerial structure in some medical schools. The most commonly introduced managerial structures are curriculum committees and sub-committees with pre-defined members and tasks [30, 31]. Hendricson [36] revealed that curriculum committees with a specified annual assignment from the Dean and those with a course review/evaluation history have a significant impact. In another study by Hendricson [38], little changes were reported in the participants’ viewpoints regarding curriculum committee tasks, mission, and impact from 1987 to 1990. Suggestions given in this study to increase the efficiency of curriculum committees comprised of restructuring committee membership (e.g., base of teaching for membership, fewer members for better group function, and increased appointment duration for members and committee chairs), giving more authority to the curriculum committee, developing clear operational guidelines for the committee, and establishing better communication between the committee and other curriculum stakeholders [38]. It can be concluded that the curriculum committee will be an appropriate managerial structure for UME curriculum management if it has clearly established plans for reviewing and evaluating educational programs and if it can establish good interpersonal communication.

Overall, reviewing papers related to UME curriculum management or monitoring revealed some practical points:

1. The subject of curriculum management is considered in accreditation standards for medical schools; for example, the WFME standard B 7.1.1 states: “The medical school must have a programme of routine curriculum monitoring of processes and outcomes” [10] and the LCME standard number ED-33 highlights the importance of institutional responsibility of medical education programs regarding curriculum management [9].
2. Having curriculum committees is a useful structure for UME curriculum management/monitoring. There is no best way to organize a curriculum committee, and each educational institution should form its own committee regarding its conditions and resources.
3. Considering the huge UME curriculum data, use of a CMS seems to be necessary. Such a system should be tailored to each institution.

## Conclusions

Medical schools can manage/monitor their UME curriculum delivery in different ways. There is no single best approach suitable for all UME programs across the world because of the differences in curricular features. A suitable managerial structure (e.g., the UME curriculum committee) with good coordination and teamwork between members and other curriculum stakeholders (faculty members, students, etc.) and technological facilities (e.g., CMS) can be helpful for every medical school in order to perform an effective and efficient UME curriculum management/monitoring. In this way, medical schools can meet accreditation standards relevant to curriculum management. Moreover, designing a system to share curriculum information between medical schools can be beneficial for UME curriculum improvement using successful experiences of different schools.

## Abbreviations

AAMC: Association of American Medical Colleges
AR: Augmented reality
CMS: Curriculum management system
CurrMIT: Curriculum management & information tool
DOTS: Directly observed treatment short-course
HCAI: Healthcare-associated infection
LCME: Liaison Committee on Medical Education
LCMS: Learning Content/Curriculum Management System
LMS: Learning management system
TB: Tuberculosis
UME: Undergraduate Medical Education
WFME: World Federation for Medical Education
